# High-throughput methodology to identify CRISPR-generated *Danio rerio* mutants using fragment analysis with unmodified PCR products

**DOI:** 10.1016/j.ydbio.2022.02.003

**Published:** 2022-02-09

**Authors:** Sarah Colijn, Ying Yin, Amber N. Stratman

**Affiliations:** Department of Cell Biology and Physiology, Washington University in St. Louis, School of Medicine, St. Louis, MO, 63110, United States

**Keywords:** Fragment analyzer, CRISPR, Zebrafish, Indels, Cilia, Genotyping

## Abstract

Targeted mutagenesis in zebrafish, fruit flies, and *C. elegans* has been significantly improved over the years through CRISPR technology. CRISPR enables researchers to efficiently examine cellular pathways by inducing small, targeted mutations *in vivo*. Though these mutations are commonly random insertions or deletions (indels), they often result in functionally disrupted alleles of a target gene if the CRISPR components are appropriately designed. However, current protocols used to identify the presence of CRISPR-generated indels are often labor intensive, time-consuming, or expensive. Here, we describe a straightforward, high-throughput method for identifying the presence of mutations by using a fragment analyzer platform which allows for DNA fragment sizing through high-resolution capillary gel-electrophoresis. Following this protocol, small indels—down to 2 base pairs—can be quickly and reliably identified, thus allowing for large-scale genotyping of newly-generated or stable mutant lines.

## 1. Introduction

The use of CRISPR technology is an efficient, effective, and increasingly popular method to generate mutant lines in zebrafish (*Danio rerio*) (Liu et al., 2019; Varshney et al., 2016). CRISPR technology utilizes the family of Cas proteins (Cas9, Cas12a/Cpf1, etc.) and guide RNAs (gRNAs) to target and cut specific DNA sequences in living cells (Wright et al., 2016). The cleaved DNA will often be improperly repaired via non-homologous end-joining, resulting in an insertion-deletion mutation (indel) and thereby disrupting normal gene transcription through frameshift, missense, or nonsense mutations (Ran et al., 2013; Tuladhar et al., 2019). Zebrafish embryos that are injected at the one-cell stage with Cas protein and an appropriate gRNA have a high probability of acquiring indels (Hoshijima et al., 2019; Varshney et al., 2015). However, the analysis, identification, and maintenance of mutant lines harboring indels generated from CRISPR protocols can be time-consuming or costly, especially when screening hundreds of samples at a time. Here, we show a method in which indels can be quickly, reliably, and affordably identified and tracked using the Agilent 5300 Fragment Analyzer, which allows for indel sizing through high-resolution capillary gel-electrophoresis. This technique is similar to indel detection by amplicon analysis (IDAA)—with comparable precision and indel analysis capabilities (Yang et al., 2015); however, the method we outline here has the added benefit of not requiring amplicon tagging.

**Fig. 1.**
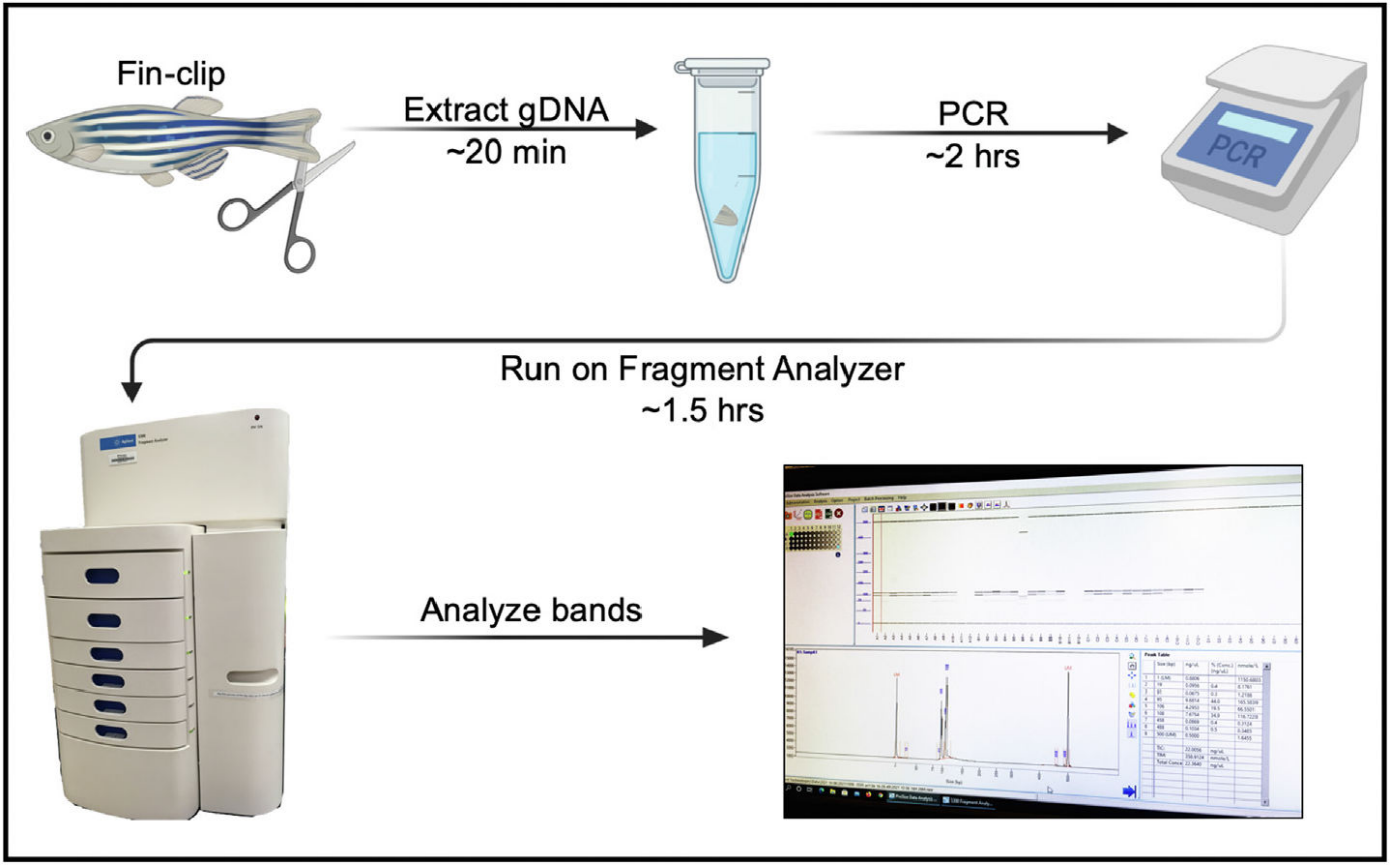
Genotyping workflow using the Agilent 5300 Fragment Analyzer. Clip a small piece of an adult zebrafish fin (or use a whole embryo) and extract the gDNA. Perform standard PCR using the gDNA and primers that flank the target region of interest. Run the PCR samples on the Agilent 5300 Fragment Analyzer, then analyze the DNA band sizes. The entire protocol—from gDNA extraction to data analysis—can be completed in 4 h (Figure was made using BioRender.com.)

In brief, DNA fragments generated from standard polymerase chain reactions (PCR) flanking the mutagenized region-of-interest are loaded and run on the fragment analyzer which then separates the DNA based on size. In our hands, the Agilent 5300 Fragment Analyzer can reliably distinguish differences in size down to 2-base pairs (bp) in resolution with no need for an added restriction enzyme digestion step and no risk of ethidium bromide toxicity, gel bleaching, or sample loss due to gel running errors. The equipment is easy to use, costs around $1 per sample, and the data is easy to interpret. The entire process—from tissue DNA extraction to indel analysis—takes approximately 4 hours to complete with minimal labor required (Fig. 1). While the fragment analyzer method requires an investment in the hardware, the efficiency of the method makes it an invaluable tool for zebrafish, fruit fly, and *C. elegans* mutagenesis screening studies and for high-throughput genotyping of stable mutations.

**Fig. 2.**
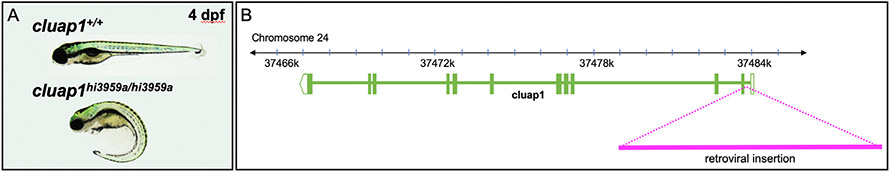
*cluap1*^*hi3959a*^ mutants have a 10 kb retroviral insertion in intron 1 of the *cluap1* locus and display body axis symmetry defects. (A) Images of control and *cluap1*^*hi3959a/hi3959a*^ homozygous mutants at 4 dpf. Mutants display body curvature, a common phenotype of *Danio rerio* lines with mutant cilia proteins (Cao et al., 2010; Li and Sun, 2011; Sullivan-Brown et al., 2008). (B) Representation of the *Danio rerio cluap1* locus. *Cluap1* is oriented on the minus strand of chromosome 24 and has 12 exons. The 10 kb retroviral insertion that was used to make the *cluap1*^*hi3959a*^ line sits within intron 1, prior to the translation start site (Li and Sun, 2011).

To illustrate the methods involved in generating and analyzing CRISPR mutants, we will describe our injection protocols and fragment analyzer workflow to generate a stable *Danio rerio* line that has a mutated cilia gene. Our goal was to specifically target the intraflagellar transport protein Cluap1, which is important for cilia biogenesis and cargo transport along the cilium (Pasek et al., 2012). The previously published *cluap1^hi3959a^* mutant line was generated using a retroviral insertion of 10,000 bp into intron 1 of *cluap1* on the minus strand of chromosome 24 (Fig. 2), thereby resulting in a zygotic null allele (Li and Sun, 2011; Sun et al., 2004). Due to the size of the retroviral insertion, we sought to generate a small, targeted *cluap1* loss-of-function allele specifically in the *cluap1* locus to decrease the possibility of genetically linked off-target effects. To create this targeted *cluap1* mutant line, we used CRISPR technology and fragment analysis to successfully identify loss-of-function indels in the *cluap1* locus. We will outline the workflow and our results here.

## 2. Materials

### 2.1. For injection

- Alt-R^®^ L.b. Cas12a crRNA [custom; IDT]
- EnGen^®^ Lba Cas12a (Cpf1) [M0653; NEB]
- 2M KCl solution*
- Phenol red solution [P0290; Sigma]
- Petri dishes, 100 mm [FB0875713; Fisher Scientific]
- Injection needles [TW100F-4; WPI; stretched using the micropipette puller]
- Egg molds (if desired)*
- Egg water*

### 2.2. For genotyping

- Agilent dsDNA 905 Reagent Kit (ladder, gel, etc.) [DNF-905-K0500; Agilent]
- 96 DeepWell Plate 1 mL [12566120; Fisher Scientific]
- 96-well 0.2 mL semi-skirted ABI Type PCR Plate, A12 cut [PR-PCR2296; MidSci]
- gDNA Extraction components [Sigma]:
  - Extraction Solution [E7526]
  - Tissue Preparation Solution [T3073]
  - Neutralization Solution B [N3910]
- PCR Primers [custom DNA oligos; IDT]
- Dream Taq^™^ Green PCR Master Mix (2x) [K1082; ThermoFisher]
- Mineral oil [M5904; Sigma]

### 2.3. For sequencing

- Phusion Hot Start II High-Fidelity PCR Master Mix [F-565; ThermoFisher]
- Zero Blunt^®^ TOPO^®^ PCR Cloning Kit for Sequencing [45-0031; Invitrogen]
- Agar plates with antibiotic*
- One Shot^™^ TOP10 Chemically Competent *E. coli* [C4040; ThermoFisher]

## 3. Equipment

- Mating Tanks
- PV820 Pneumatic PicoPump
- Model P-1000 Flaming/Brown Micropipette Puller
- Agilent 5300 Fragment Analyzer (with the 48-capillary Short Array)
- Nikon SMZ18 Stereo Microscope

## 4. Methods

For the design and acquisition of CRISPR reagents, online databases such as CHOPCHOP and CRISPRscan.org are easy to use to generate gRNAs that target a gene-of-interest (Labun et al., 2019; Moreno-Mateos et al., 2015), while companies such as Integrated DNA Technologies (IDT) provide custom RNA services to construct ready-to-inject gRNAs. Cas proteins such as Cas9 and Cas12a/Cpf1 can be inexpensively purchased from many scientific reagent companies like New England Biolabs (NEB). These ready-to-use and affordable services render in-lab construction of gRNAs or Cas mRNAs unnecessary, and for these reasons were used in our workflow.

### 4.1. Design the gRNAs

1. Use the CHOPCHOP online database (https://chopchop.cbu.uib.no) to identify gRNAs for your gene-of-interest. Enter in your target gene and organism, choose the CRISPR method, and select the intended outcome (knock-out, knock-in, etc.). Browse the resulting gRNA suggestions to find your ideal target region and sequence.
  - To create a null *cluap1* gene in *Danio rerio*, we selected *cluap1* as the target, *Danio rerio* (danRer11/GRCz11) for the organism, CRISPR/Cpf1 as the method, and knock-out for the outcome. We chose to use Cas12a/Cpf1 due to reports that it is more precise than Cas9 and it can be injected with CRISPR RNA (crRNA) without an additional trans-activating crRNA (tracrRNA) (Fernandez et al., 2018; Moreno-Mateos et al., 2017).
  - We selected a target sequence whose PAM cleavage site targets the translation start site (ATG triplet) of *cluap1* in exon 2.
    Target sequence: 5’-TTTACTGGATTAAAGGACAGCAATCAT-3’
2. If purchasing custom crRNAs from IDT, remove the PAM site (TTTN) from the 5’ region of the target sequence before entering the DNA sequence into the IDT order window. Once ordered and received, dilute the lyophilized Alt-R^®^ Cas12a crRNAs in nuclease-free water to make a 100 μM solution.

### 4.2. Inject the CRISPR reagents into zebrafish embryos

**Fig. 3.**
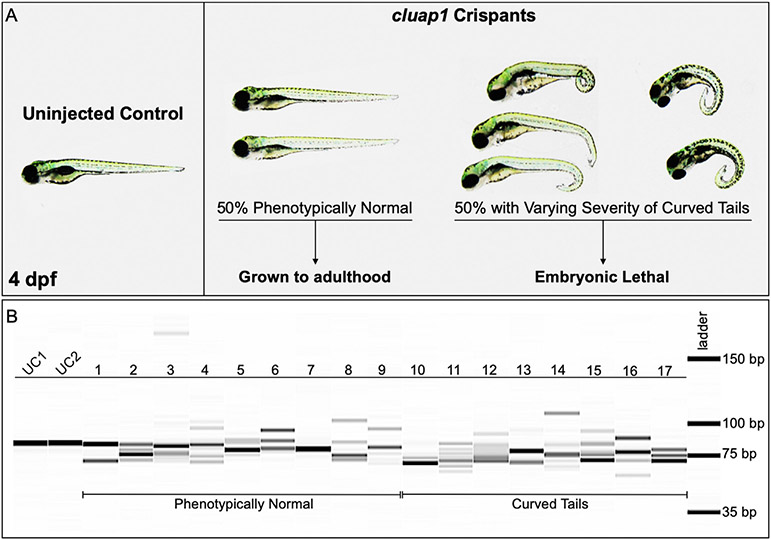
*cluap1* crispants display varying body curvature severity and successful CRISPR targeting. (A) Images of uninjected control (left) and *cluap1* crispant embryos (right) at 4 dpf. Approximately 50% of the crispants developed the tail curvature and embryonic lethality indicative of homozygous mutations in cilia proteins (Cao et al., 2010; Li and Sun, 2011; Sullivan-Brown et al., 2008), though the severity of the tail curvature varied. The remaining phenotypically normal survivors were grown to adulthood (F0 generation) to use for breeding stable mutant lines. (B) Crispants were collected at 4 dpf and genotyped for the presence of indels with the fragment analyzer workflow. Image of the fragment analyzer gel output data is shown. The first two lanes (UC1, UC2) are uninjected sibling controls. Lanes 1–9 are crispants that appear phenotypically normal. Lanes 10–17 are crispants that display tail curvature. Only 1 out of 35 genotyped crispants displayed a single wildtype band (Lane 7), indicating a success rate of 97% (34/35) for indel generation.

1. Set up zebrafish breeders in divided mating tanks the night before injections. Choose breeder strains that assist in your downstream applications. For example, if you intend to analyze mutant zebrafish using a particular cell-type-specific fluorescent transgenic line, you can inject directly into that line. In contrast, if you expect to cross the mutant zebrafish to a variety of transgenes, you may wish to start with wildtype breeders.
  - We used AB wildtype breeders for our *cluap1* CRISPR targeting.
2. The next morning, prepare the injection solution.
  - Recipe to make 10 μL of Cas12a/crRNA injection stock solution:
    1.2 μL crRNA (100 μM stock).
    1.95 μL 2M KCl.
    4.85 μL H_2_O
    1 μL Lba Cas12a (100 μM stock; add last to prevent precipitation).
  - Incubate solution at 37 °C for 10 min.
  - Add 1 μL phenol red.
  - Leave injection mix at room temperature prior to injecting.
3. Pull the dividers from the zebrafish mating tanks and collect the embryos immediately upon laying. Prepare the needle and injection rig. Align the eggs for injection in the egg mold and inject 1 nL of the stock solution into each embryo’s single cell before they divide. The eggs will begin dividing around 30–45 min after laying (Kimmel et al., 1995).
4. After injecting, place the embryos into a 28 °C incubator and allow them to grow. If targeting efficiency is expected to be low, or if the mutation is not expected to cause embryonic lethality, then incubate the embryos at 34°C for 4 h before moving to 28 °C for increased Cas12a activity (Moreno-Mateos et al., 2017).
  - Homozygous *cluap1*^*hi3959a*^ mutants are embryonic lethal; therefore, we sought to avoid optimal conditions for Cas12a activity as this could affect the survival of successfully targeted CRISPR mutants. In our hands, avoiding the 34 °C incubation allowed for sufficient survival while simultaneously inducing a variety of CRISPR-generated mutations in 97% of injected embryos (Fig. 3).
5. Grow the surviving embryos to adulthood. Concentrations of crRNAs may need to be adjusted if survival is low. Collect a small number of the embryos (10–20) for genotyping to confirm that successful CRISPR targeting has occurred.
  - For our *cluap1* crRNA/Cas12a injections, 85% of embryos survived to 1 day post fertilization (dpf). Approximately 50% of the survivors developed the tail curvature and embryonic lethality indicative of homozygous mutations in cilia proteins (Fig. 3a) (Cao et al., 2010; Li and Sun, 2011; Sullivan-Brown et al., 2008). The remaining survivors were grown to adulthood (F0 generation) and used for breeding stable mutant lines.
  - Technical Note: F0 survival could vary widely depending on the targeted gene of interest, the crRNA target region, the concentration of crRNA, the off-target effects of the CRISPR reagents, the quality of the embryo injection technique, and the activity level of Cas12a. Troubleshooting these factors may be required to acquire viable F0 adults.

### 4.3. Genotype the F0 generation using fragment analysis

**Fig. 4.**
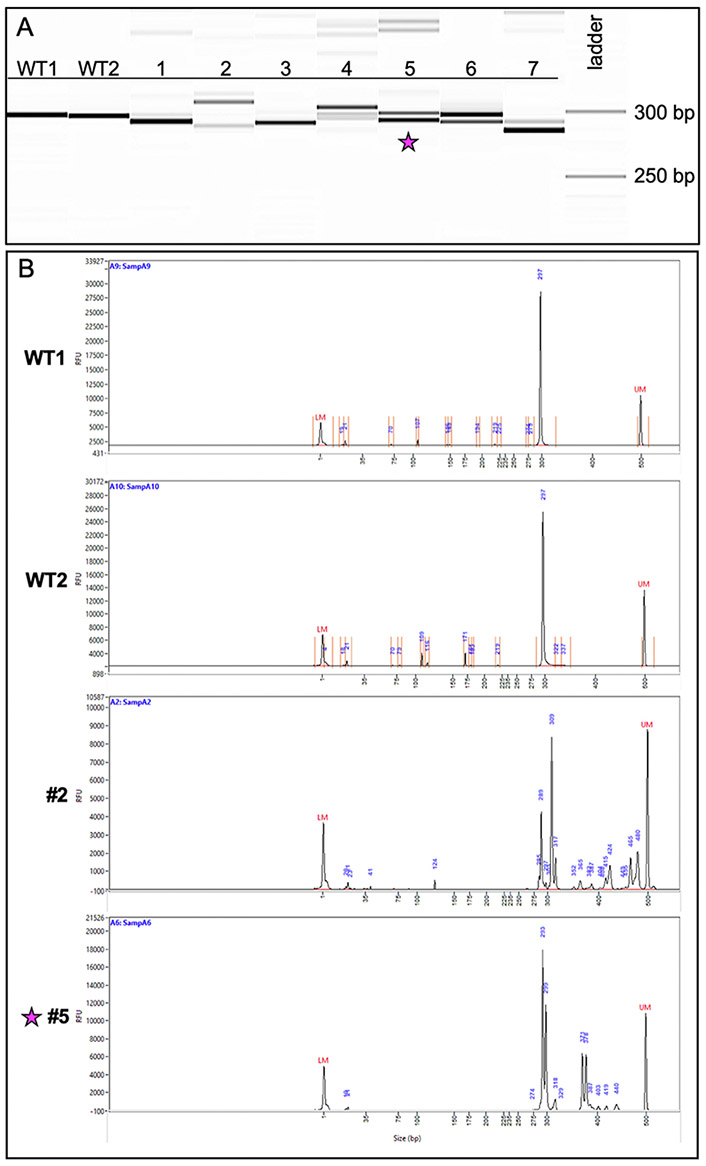
Genotyping of CRISPR-injected F0 adults reveals a variety of indel species. (A) Image of the fragment analyzer gel output data. The first two lanes (WT1, WT2) are wildtype fish and display a band size of 297 bp. Lanes 1–7 are fish from the CRISPR-injected adult F0 generation. (B) Image of the electropherograms for samples WT1, WT2, 2, and 5. The taller peaks correspond to the more intense bands on the gel. The peaks for #2 (289 bp and 309 bp) suggest the presence of two primary amplicons present in a high percentage of the gDNA: one with a potential 8-bp indel and the other a potential 12-bp indel. Less prevalent amplicons exist at larger bp sizes ranging between 317 and 480 bp. The peaks for #5 (293 bp and 299 bp) suggest the presence of a predominant 6-bp indel. Fish #5 (indicated with a star) was successfully bred and used to establish a stable mutant line.

1. Choose a sizing kit from Agilent to assess the type of mutation you seek to analyze. The optimal kit for identifying small indels is the dsDNA 905 Reagent kit, which allows the Agilent 5300 Fragment Analyzer to resolve DNA sizes between 1 and 500 bp. If you select the dsDNA 905 Reagent kit, design primers that produce an amplicon of greater than 35 bp and less than 500 bp.
  - Technical Note: Agilent notes that smaller amplicon sizes will result in improved indel resolution (Pocernich et al., 2019). We have also observed this phenomenon (SFig. 1). However, small amplicons (50–150 bp) may not accurately detect larger indel species, especially if the primer binding site becomes mutated. We recommend analyzing the F0 generation with a primer pair that generates a larger 200–300 bp amplicon, either individually or in tandem with a primer pair that generates a smaller amplicon. Once an indel has been successfully identified, optimized primers flanking indels of choice can be generated to propagate and maintain stable lines.
2. Fin-clip adult F0 zebrafish and extract the genomic DNA (gDNA). Alternatively, extract gDNA from the small number of F0 embryos to test for successful CRISPR/Cas12a targeting and DNA cleavage.
3. Using primers that flank the mutated region, perform PCRs on the extracted gDNA for each sample (10 μL is a sufficient reaction volume).
  - PCR primers were designed to flank the translation start site of *cluap1* to create a predicted 266 bp amplicon and a predicted 74 bp amplicon. We recommend designing primers that overlap exons if possible as introns may accumulate benign single nucleotide polymorphisms (SNPs) across multiple generations, thus risking primer binding affinity.
    Primers for 266 bp amplicon: Forward 5’-TGAGGCTAACCACATTTTCCA-3’; Reverse 5’-GGGGTCACTTACTTCTCAGATCTCTA-3’
    Primers for 74 bp amplicon: Forward 5’-GGTGACAGTTGTGTGTTTACTGG-3’; Reverse 5’-GGGGTCACTTACTTCTCAGATCTCTA-3’
4. Load PCR reactions into 96-well plates that are approved for use on the Agilent 5300 Fragment Analyzer. Add 14 μL TE Buffer to each well for a final volume of 24 μL per well. Depending on the capillary array that is installed in the machine (12-, 48-, or 96-capillary arrays), fill any remaining empty wells with 24 μL TE Buffer. In the last well (A12 for 12-capillary, D12 for 48-capillary, or H12 for 96-capillary), add 24 μL of the sizing ladder. (We have found that loading between 15 and 30 μL in each well does not affect the output of the run, so 24 μL is simply a suggestion from Agilent.)
5. Spin down the samples in the 96-well plate. Add a drop of mineral oil to the top of each sample if you intend to use the reactions for a downstream application or if the reactions will be sitting uncovered for a prolonged period. This prevents sample evaporation.
6. Prepare or refresh the gel, inlet buffer plate, capillary storage plate, capillary conditioning solution, and marker plate as directed by the Agilent instructions. The Agilent Fragment Analyzer protocol is straightforward, standardized, and optimized and should not need to be altered between users. The reagents must be manufactured by Agilent and not substituted by reagents from a secondary source to prevent damaging the sensitive capillary equipment.
7. Load the sample plate and all other reagent plates into their appropriate drawers, then add your samples to the ‘Queue’. Use the standard default run method that matches your selected analysis kit. The default method should take 80–90 min per run. Depending on the capillary array installed in the machine, one run can accommodate 12, 48, or 96 samples. If multiple runs are needed, other sample plates can be loaded, added to the ‘Queue’, and will run in the order they are queued. This allows for consecutive runs without the need for monitoring or manipulating the machine between samples. The results will automatically save to the computer after each run.
8. Open the ProSize Data Analysis Software to view the results. The results will be displayed as a gel image (Figs. 3b and 4a) with electropherograms (Fig. 4b) and a “Peak Table” (as seen in Fig. S2). The electropherograms are labeled with DNA band sizes. The gel image also displays DNA band sizes when you scroll the mouse pointer over the band. If the bands appear to be faint, increase the contrast by using the scroll bar to the right of the gel image. The “Peak Table” lists the sizes of detectable bands with predicted band concentrations (in ng/μL) and the ratio of each band to total sample DNA (in percentages).
  - After following this workflow (Fig. 1), our results from F0 crispant embryos displayed a variety of indels—indicating successful targeting (Fig. 3b)—while fin clips of adult F0 fish displayed wildtype bands and indel species between 6 and 20 bp in size localized around the CRISPR target site (Fig. 4). Note that a highly prevalent 6-bp indel can be visualized via fragment analysis for the adult F0 fish #5.

### 4.4. Grow and genotype the F1 generation using fragment analysis

**Fig. 5.**
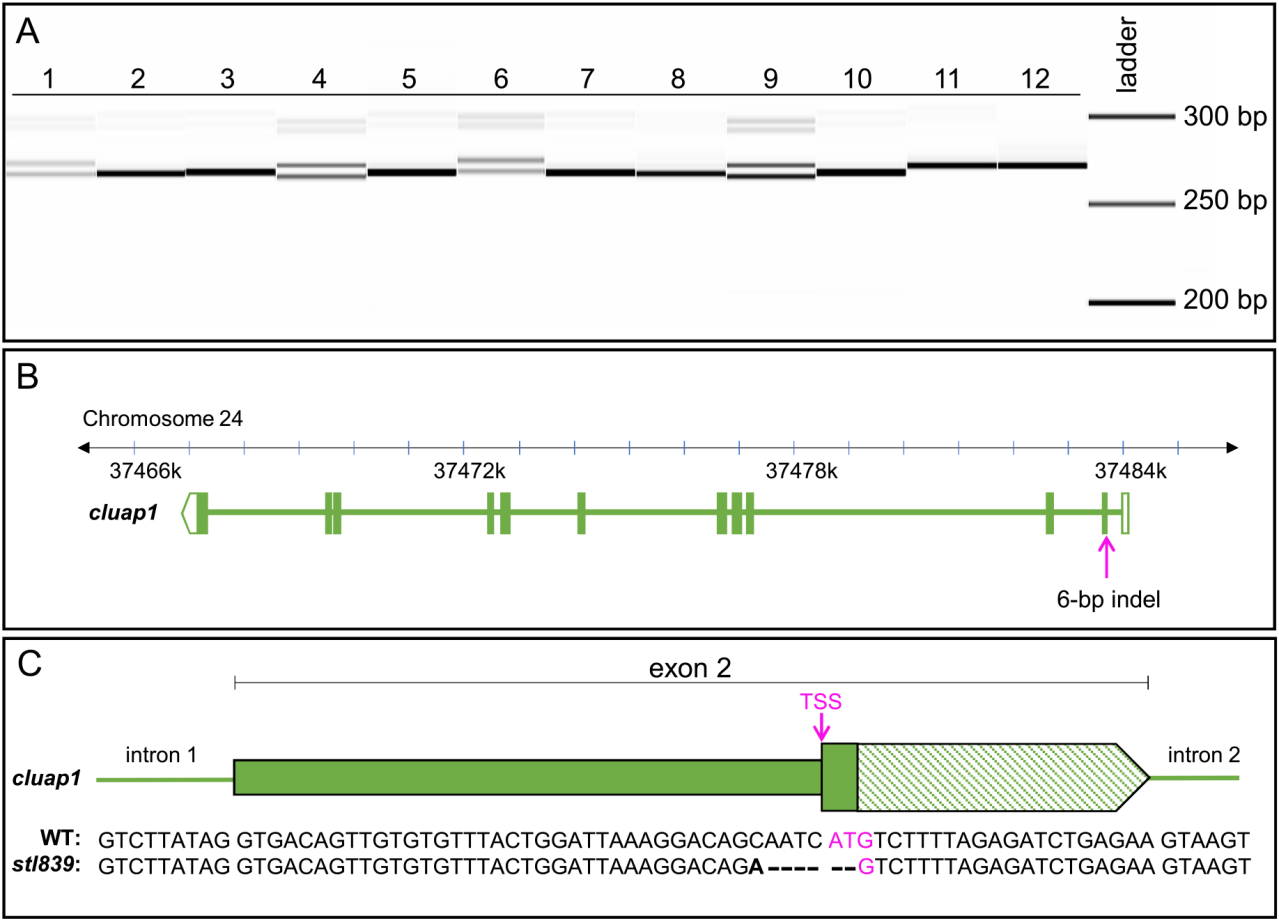
Genotyping of embryos from the F1 generation reveals germline transmission of the 6-bp indel, which is confirmed via sequencing. (A) Image of the fragment analyzer gel output data. Lanes 1–12 are F1 embryos collected from a cross between F0 fish #5 and a wildtype AB zebrafish breeder. Single bands (Lanes 2, 3, 5, 7, 8, 10, 11, and 12) represent wildtype amplicons. The doublets (Lanes 1, 4, 6, and 9) represent heterozygous carriers of the identified 6-bp indel. (The less intense bands visible at 300 bp are PCR artifacts and are not present with the use of an alternate primer pair.) (B) Representation of the *Danio rerio cluap1* locus with the location of the 6-bp indel. The CRISPR target site is in the TSS of *cluap1* in exon 2. (C) Representation of exon 2 with the sequencing results for the WT and mutant alleles. The bold letter indicates a single bp change, the dashes indicate bp deletions, and the magenta indicates the TSS. The 6-bp indel allele, now referred to as *cluap1*^*stl839*^, disrupts the TSS, leading to a predicted loss-of-function *cluap1* allele. TSS: Translation Start Site (ATG triplet).

1. Outcross the F0 adults to a wildtype breeder. Alternatively, F0 fish can be outcrossed to a preselected transgenic line if desired.
  - Healthy F0 adults were outcrossed with AB wildtype fish. Approximately 50% of surviving F0 adults were fertile, and 25% of those breeders were able to transmit one or multiple indel variations to progeny. These rates will vary between experiments depending on the target gene, target region, and the level of Cas12a activity.
2. From the outcross, collect approximately 20–30 embryos at 2–3 dpf for genotyping via fragment analysis to confirm germline transmission of an indel (F1 generation). If an indel (or multiple) are present in the F1 generation, grow the remaining embryos to adulthood.
  - Technical Note: If many F0 breeders produce embryos, genotype the embryos in bulk (pooling gDNA from 10 embryos per PCR reaction) to determine which F0 outcross produces germline transmission. The Agilent 5300 Fragment Analyzer can detect low concentrations of DNA bands that represent less than 5% of the total DNA sample (SFig. 2). Therefore, indels that appear at low prevalence have a high probability of being detected in pooled samples. Once an F0 founder is identified, proceed with genotyping individual F1 embryos from the outcross to further assess types of indels present.
  - Fragment analysis revealed the presence of a 6-bp indel in the F1 progeny of F0 fish #5 (Fig. 5a).

### 4.5. Sequence gDNA from F1 embryos

1. Use gDNA from the F1 embryos carrying an indel in the previous step for Sanger sequencing. Alternatively, you can fin-clip the F1 generation after they have grown to adulthood to identify indel carriers and use this gDNA for TOPO^®^ cloning and Sanger sequencing.
2. Using the previously designed PCR primers, perform PCR with a proofreading polymerase for rapid TOPO^®^ PCR cloning. Follow the instructions from the TOPO^®^ cloning kit to insert the PCR products into the TOPO^®^ vector, then transform into competent *E. coli*.
3. Send the agar plates with colonies for Direct Colony Sequencing. Sequence 8–12 colonies per embryo. (Direct Colony Sequencing saves time and reagents since plasmid miniprepping is unnecessary. However, if the TOPO^®^ vector will be used in other downstream applications, then miniprep the plasmids and send purified DNA for sequencing.)
  - Our sequencing results revealed that the identified indel is the result of a 6-bp deletion plus a single bp change. Additionally, sequencing confirmed that the indel occurred at the CRISPR target region within the *cluap1* translation start site so that the resulting frameshift disrupted the ATG triplet (Fig. 5b and c). This allele is now referred to as *cluap1*^*stl839*^.
  - Technical Note: If the user prefers another method of Sanger sequencing, then the sequencing step can be altered to accommodate other protocols without affecting subsequent steps.

### 4.6. Genotype F1 adult breeders and analyze homozygous mutant embryos

**Fig. 6.**
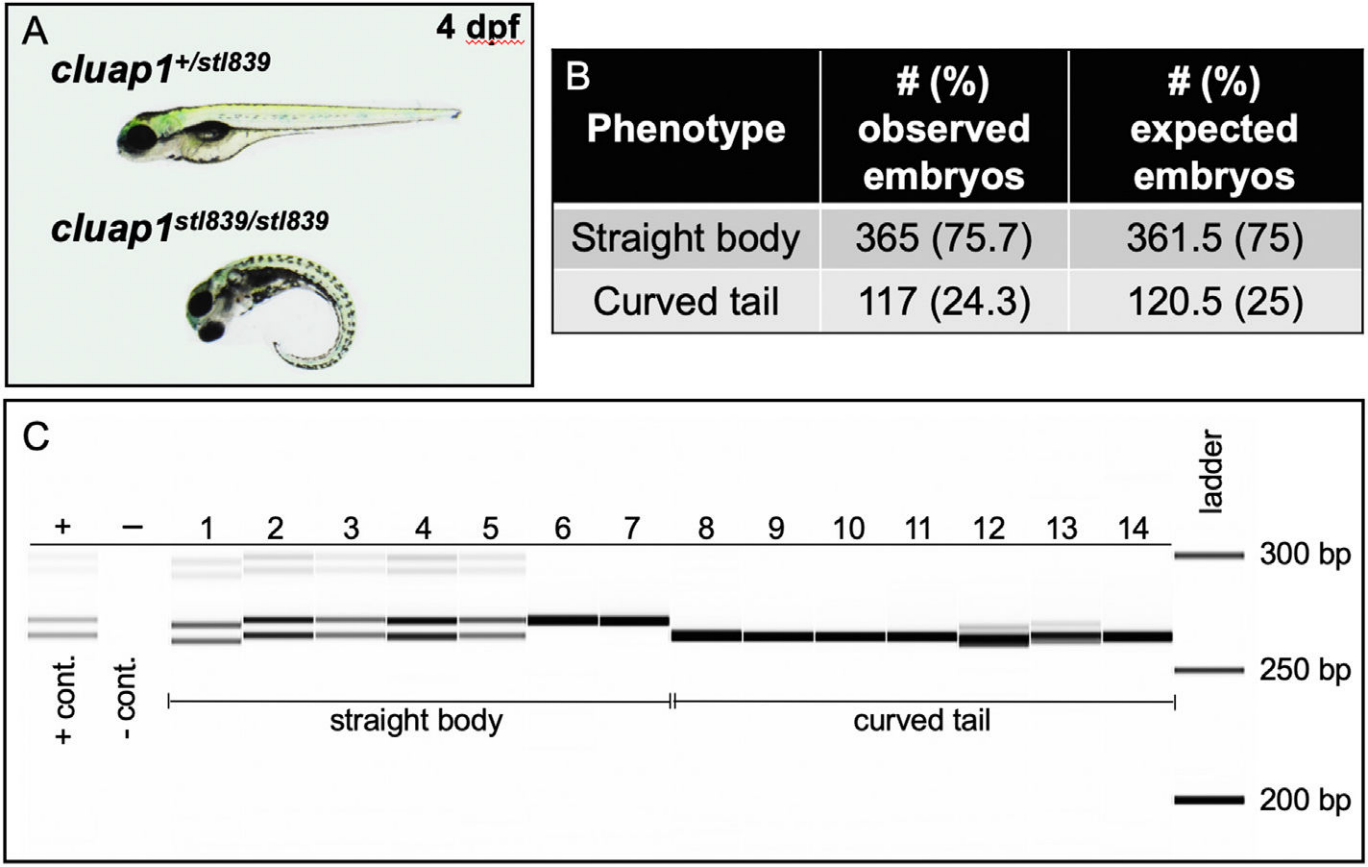
*Cluap1*^*stl839*^ mutants display body axis symmetry defects that are inherited at the Mendelian ratio and correspond to homozygosity of the mutant allele. (A) Representative images of control sibling and *cluap1*^*stl839/stl839*^ homozygous mutants at 4 dpf. Mutants display body curvature. (B) This curvature is present in 24.3% of total embryos, which suggests Mendelian inheritance of the mutant allele. (Statistics: Chi-square, observed vs. expected, p = 0.75). (C) Image of the fragment analyzer gel output data. Lane ‘+’ is the positive heterozygous control for the 6-bp indel; lane ‘−‘ is the negative control. Lanes 1–14 are F2 embryos bred from an incross between F1 adult *cluap1*^*stl839/*+^ carriers with the 6-bp indel. Lanes 1–7 represent embryos that look phenotypically normal. There is a combination of heterozygous (1–5) and homozygous wildtype (6, 7) alleles in the phenotypically normal group. Lanes 8–14 represent embryos with body curvature. All embryos with curved bodies genotype as homozygous mutants. Notice the slight variability between the sizing of bands in Lanes 1 and 12 compared to similar genotypes. Since each lane is run independently through an individual single capillary, there is a possibility for small sizing shifts between consecutive wells.

1. After the FI population is grown to adulthood, genotype using the fragment analysis workflow to identify heterozygous carriers of the indel. Incross heterozygous individuals and analyze the progeny (F2 generation) for mutant phenotypes.
  - Our F1 *cluap1*^*stl839/+*^ carriers were in-crossed, and their progeny (F2 generation) were analyzed for gross phenotypes associated with cilia mutations. The embryos displayed tail curvature (body axis symmetry defects) that are prevalent in homozygous cilia mutants (Cao et al., 2010; Li and Sun, 2011; Sullivan-Brown et al., 2008). The tail curvature was present at Mendelian ratios (Fig. 6a and b) and was consistent with phenotypes noted in the F0 injected crispants (Fig. 3a).
  - Technical Note: For analysis of zygotic mutant phenotypes, we recommend growing mutant lines out to at least the F3/F4 generation to ensure that the newly generated indel is stable and continues to segregate with observed phenotypes.
2. Genotype the embryos using the fragment analysis workflow outlined above.
  - Our F2 generation embryos that displayed curved tails were homozygous *cluap1s*^*stl839/stl839*^ mutants (Fig. 6c).
  - A phenotypic complementation test between the *cluap1*^*stl839/+*^ and the *cluap1*^*hi3959a/+*^ mutant lines resulted in progeny with curved tails inherited at Mendelian ratios (SFig. 3). This suggests that the novel *cluap1*^*stl839*^ indel results in a functionally null allele like the *cluap1*^*hi3959a*^ line.

## 5. Discussion

We have successfully identified a CRISPR-generated *Danio rerio* cilia mutant line using fragment analysis. The Agilent 5300 Fragment Analyzer was able to consistently resolve the introduced and isolated 6-bp indel. The workflow presented here demonstrates that current CRISPR mutagenesis screening assays can be streamlined through the use of a fragment analyzer that separates unmodified PCR products based on size. The consistency of a tool such as fragment analysis can provide significant workflow gains—including decreased costs, improved resolution of indels, increased automation, and quick acquisition of results—for labs or departments that need a high-throughput method to identify or genotype indels.

Benefits of the Agilent 5300 Fragment Analyzer include resolution of differing DNA fragment sizes down to 2 bp and the ability to genotype and detect indels from low concentrations of input DNA—between 0.5 and 50 ng/uL (Pocernich et al., 2019). Faint bands that would be undetectable or unresolvable using standard gel-electrophoresis equipment can be identified with the high contrast detection limits of the fragment analyzer (SFig. 2). Furthermore, each well runs as a separate electrophoresis assay. The capillaries are individual entities that pull up sample from a single well, ensuring that there is no chance for contamination from nearby wells due to spilling or mixing of samples. During data analysis, you can also select individual wells to view in the final gel image; the software will then compile them without the issues associated with “cropping” gel images.

The use of fragment analysis provides a wide range of utility for both indel identification and indel tracking. Other popular methods of indel identification include sequence decomposition software tools such as Tracking of Indels by Decomposition (TIDE) and Inference of CRISPR Edits (ICE) which use Sanger sequencing data to determine indel sizes, sequences, and frequency post hoc (Brinkman et al., 2014; Hsiau et al., 2019). While these are powerful tools for identification of indel species, they are less suitable for maintenance of a stable mutant line or genotyping experimental samples, as they require a Sanger sequencing step which can be expensive or time-consuming. Other common genotyping methods that are faster and less expensive than Sanger sequencing—such as High Resolution Melting Analysis (HRMA), T7 Endonuclease 1 mismatch detection (T7E1), and restriction fragment length polymorphism (RFLP) analysis—may be suitable for tracking indels that have already been identified, but are suboptimal for initial identification of indels as they cannot predict indel size. Furthermore, they are sensitive to polymorphisms that may be gained or lost between generations—thus frequently require re-optimization—and are less reliable for tracking large indels (Kim et al., 2014; Parant et al., 2009; Sentmanat et al., 2018; Zischewski et al., 2017). Additionally, RFLP is limited by the variety of restriction enzyme cleavage sites present, whereas T7E1 cannot be used to distinguish between homozygous mutant versus homozygous wildtype genotypes. In contrast, use of a fragment analyzer can predict indel size, indel frequency, track stable indels, and distinguish homozygous and heterozygous genotypes with high sensitivity. While a sequencing step is still required to confirm the nature of an indel species, the utility of the fragment analyzer makes it a streamlined option compared to other individual genotyping methods.

While there are many benefits to using fragment analysis, there are a few drawbacks to the Agilent 5300 Fragment Analyzer and other fragment analyzer systems that should be considered. Since the samples are run separately through individual capillaries, the same DNA bands can appear as shifted up or down in size by a few base pairs between samples or runs. Thus, comparisons between consecutively run wells are not perfect, and one can expect to see one to two base pairs size difference between the same band in one well versus another. We have calculated the accuracy and precision of the Agilent 5300 Fragment Analyzer using two amplicon sizes with the *cluap1*^*stl839*^ indel and have found that sizing precision can improve with the use of smaller amplicons (STable 1). Despite this, distinguishing between homozygous wildtype and homozygous mutant samples can be difficult for small indels when considering the sizing variation of each well. To address this, we show that adding a small concentration of PCR product from a heterozygous sample to the unknown PCR samples can resolve this issue. The intense band from a homozygous PCR product will align with either the faint wildtype or mutant bands of the heterozygous PCR sample and thus rectify the capillary-dependent sizing issues (SFig. 4).

Further, depending on how precisely the ladder runs, the whole array can run the DNA at modestly different sizes than expected. For example, we could expect a band size at 266 bp while the fragment analyzer may mark it as a 290 bp amplicon. In our hands, the most common shift errors are a size increase of up to 20% from the expected size uniformly across an entire experimental run (STable 1). The use of a positive control helps mitigate this incongruity; however, this can be an issue if the goal of an experiment is to determine the exact size of an unknown amplicon without additional sequencing or analysis. If this is the case, we would not suggest using the Agilent 5300 Fragment Analyzer.

Despite this issue, the differences in size between two bands appearing in the same well are uniform. Specifically, a heterozygous zebrafish with a wildtype and mutant band will consistently show the same size difference between the two bands, especially with the use of smaller amplicons (STable 1). In relationship to our workflow and the novel mutant generated here—regardless of the running size of the wildtype band, the mutant band will consistently be 6 bp smaller. Therefore, if the end goal of use is determining relative differences in amplicon sizes, as described here for identification and genotyping of CRISPR-generated indels, the fragment analysis platform has exceptional utility. These pros and cons of the fragment analyzer equipment should be considered before investing in the hardware.

## 6. Recipes

### 6.1. 2M KCl solution (15 mLs)

1. Dissolve 22.4 mg KCl in 5 mL H_2_O.
2. Fill to 15 mL with H_2_O.

### 6.2. Egg mold (1 mold)

1. Combine 0.5 g agarose [15510-027; Invitrogen] with 25 mL egg water.
2. Microwave until agarose is dissolved.
3. Pour into a petri dish lid and place the plastic egg mold [FM-200; IBI Scientific] on top.
4. Once solidified, remove the plastic mold, cover with egg water, and store at 4 °C.

### 6.3. Egg water (20 L)

1. Combine 1.25 g Sea Salt, 20 mL 0.1% Methylene Blue, and 20 L H_2_O.

### 6.4. Agar plates with antibiotic (15–20 plates)

1. Combine 6.9 g Bacteriological Agar [IB49171; IBI Scientific] with 7.5 g Lennox Broth [IB49111; IBI Scientific] and fill to 300 mL with H_2_O. Mix well.
2. Autoclave. While warm, add 300 μL Ampicillin stock (100 mg/mL) and swirl.
3. Pour into petri dishes until bottom is covered. Allow agar to solidify then store at 4 °C.

## Supplementary Material

Supplmental Material

## Footnotes

* See Recipes.

## References

[R1] Brinkman EK, Chen T, Amendola M, van Steensel B, 2014. Easy quantitative assessment of genome editing by sequence trace decomposition. Nucleic Acids Res. 42 e168–e168. 25300484 10.1093/nar/gku936 PMC4267669

[R2] Cao Y, Park A, Sun Z, 2010. Intraflagellar transport proteins are essential for cilia formation and for planar cell polarity. J. Am. Soc. Nephrol 21, 1326–1333. 20576807 10.1681/ASN.2009091001 PMC2938599

[R3] Fernandez JP, Vejnar CE, Giraldez AJ, Rouet R, Moreno-Mateos MA, 2018. Optimized CRISPR-Cpf1 system for genome editing in zebrafish. Methods 150, 11–18. 29964176 10.1016/j.ymeth.2018.06.014 PMC7098853

[R4] Hoshijima K, Jurynec MJ, Klatt Shaw D, Jacobi AM, Behlke MA, Grunwald DJ, 2019. Highly efficient CRISPR-cas9-based methods for generating deletion mutations and F0 embryos that lack gene function in zebrafish. Dev. Cell 51, 645–657 e644. 31708433 10.1016/j.devcel.2019.10.004 PMC6891219

[R5] Hsiau T, Conant D, Rossi N, Maures T, Waite K, Yang J, Joshi S, Kelso R, Holden K, Enzmann BL, Stoner R, 2019. Inference of CRISPR Edits from sanger trace data. bioRxiv, 251082. 10.1089/crispr.2021.0113 35119294

[R6] Kim JM, Kim D, Kim S, Kim J-S, 2014. Genotyping with CRISPR-Cas-derived RNA-guided endonucleases. Nat. Commun 5, 3157. 24445736 10.1038/ncomms4157

[R7] Kimmel CB, Ballard WW, Kimmel SR, Ullmann B, Schilling TF, 1995. Stages of embryonic development of the zebrafish. Dev. Dynam 203, 253–310. 10.1002/aja.1002030302 8589427

[R8] Labun K, Montague TG, Krause M, Torres Cleuren YN, Tjeldnes H, Valen E, 2019. CHOPCHOP v3: expanding the CRISPR web toolbox beyond genome editing. Nucleic Acids Res. 47, W171–W174. 31106371 10.1093/nar/gkz365 PMC6602426

[R9] Li J, Sun Z, 2011. Qilin is essential for cilia assembly and normal kidney development in zebrafish. PLoS One 6, e27365. 22102889 10.1371/journal.pone.0027365 PMC3216947

[R10] Liu K, Petree C, Requena T, Varshney P, Varshney GK, 2019. Expanding the CRISPR toolbox in zebrafish for studying development and disease. Front. Cell Dev. Biol 7. 10.3389/fcell.2019.00013 PMC6409501 30886848

[R11] Moreno-Mateos MA, Fernandez JP, Rouet R, Vejnar CE, Lane MA, Mis E, Khokha MK, Doudna JA, Giraldez AJ, 2017. CRISPR-Cpf1 mediates efficient homology-directed repair and temperature-controlled genome editing. Nat. Commun 8, 2024–2024. 29222508 10.1038/s41467-017-01836-2 PMC5722943

[R12] Moreno-Mateos MA, Vejnar CE, Beaudoin J-D, Fernandez JP, Mis EK, Khokha MK, Giraldez AJ, 2015. CRISPRscan: designing highly efficient sgRNAs for CRISPR-Cas9 targeting in vivo. Nat. Methods 12, 982–988. 26322839 10.1038/nmeth.3543 PMC4589495

[R13] Parant JM, George SA, Pryor R, Wittwer CT, Yost HJ, 2009. A rapid and efficient method of genotyping zebrafish mutants. Dev. Dynam 238, 3168–3174. 10.1002/dvdy.22143 PMC3888828 19890916

[R14] Pasek RC, Berbari NF, Lewis WR, Kesterson RA, Yoder BK, 2012. Mammalian Clusterin associated protein 1 is an evolutionarily conserved protein required for ciliogenesis. Cilia 1, 20–20. 23351563 10.1186/2046-2530-1-20 PMC3556011

[R15] Pocernich C, Luttgeharm K, Wong K-S, 2019. Highly Resolved Separation of DNA Fragments on the Agilent 5200 Fragment Analyzer System (Application Note #5994-0517EN). Agilent Technologies, Inc.. Retrieved from Agilent.com. https://www.agilent.com/cs/library/applications/application-dna-fragment-separation-fragment-analyzer-5994-0517en-agilent.pdf

[R16] Ran FA, Hsu PD, Wright J, Agarwala V, Scott DA, Zhang F, 2013. Genome engineering using the CRISPR-Cas9 system. Nat. Protoc 8, 2281–2308. 24157548 10.1038/nprot.2013.143 PMC3969860

[R17] Sentmanat MF, Peters ST, Florian CP, Connelly JP, Pruett-Miller SM, 2018. A survey of validation strategies for CRISPR-cas9 editing. Sci. Rep 8, 888. 29343825 10.1038/s41598-018-19441-8 PMC5772360

[R18] Sullivan-Brown J, Schottenfeld J, Okabe N, Hostetter CL, Serluca FC, Thiberge SY, Burdine RD, 2008. Zebrafish mutations affecting cilia motility share similar cystic phenotypes and suggest a mechanism of cyst formation that differs from pkd2 morphants. Dev. Biol 314, 261–275. 18178183 10.1016/j.ydbio.2007.11.025 PMC2453220

[R19] Sun Z, Amsterdam A, Pazour GJ, Cole DG, Miller MS, Hopkins N, 2004. A genetic screen in zebrafish identifies cilia genes as a principal cause of cystic kidney. Development 131, 4085–4093. 15269167 10.1242/dev.01240

[R20] Tuladhar R, Yeu Y, Tyler Piazza J, Tan Z, Rene Clemenceau J, Wu X, Barrett Q, Herbert J, Mathews DH, Kim J, Hyun Hwang T, Lum L, 2019. CRISPR-Cas9-based mutagenesis frequently provokes on-target mRNA misregulation. Nat. Commun 10, 4056. 31492834 10.1038/s41467-019-12028-5 PMC6731291

[R21] Varshney GK, Carrington B, Pei W, Bishop K, Chen Z, Fan C, Xu L, Jones M, LaFave MC, Ledin J, Sood R, Burgess SM, 2016. A high-throughput functional genomics workflow based on CRISPR/Cas9-mediated targeted mutagenesis in zebrafish. Nat. Protoc 11, 2357–2375. 27809318 10.1038/nprot.2016.141 PMC5630457

[R22] Varshney GK, Pei W, LaFave MC, Idol J, Xu L, Gallardo V, Carrington B, Bishop K, Jones M, Li M, Harper U, Huang SC, Prakash A, Chen W, Sood R, Ledin J, Burgess SM, 2015. High-throughput gene targeting and phenotyping in zebrafish using CRISPR/Cas9. Genome Res. 25, 1030–1042. 26048245 10.1101/gr.186379.114 PMC4484386

[R23] Wright Addison V., Nuñez James K., Doudna Jennifer A., 2016. Biology and applications of CRISPR systems: harnessing nature’s toolbox for genome engineering. Cell 164, 29–44. 26771484 10.1016/j.cell.2015.12.035

[R24] Yang Z, Steentoft C, Hauge C, Hansen L, Thomsen AL, Niola F, Vester-Christensen MB, Frödin M, Clausen H, Wandall HH, Bennett EP, 2015. Fast and sensitive detection of indels induced by precise gene targeting. Nucleic Acids Res. 43 e59–e59. 25753669 10.1093/nar/gkv126 PMC4482057

[R25] Zischewski J, Fischer R, Bortesi L, 2017. Detection of on-target and off-target mutations generated by CRISPR/Cas9 and other sequence-specific nucleases. Biotechnol. Adv 35, 95–104. 28011075 10.1016/j.biotechadv.2016.12.003

