# Supplementary material for: High-throughput methodology to identify CRISPR-generated Danio rerio mutants using fragment analysis with unmodified PCR products
Source: Dev Biol. Author manuscript; Available in PMC 2023 May 8. (PMC10164608; doi:10.1016/j.ydbio.2022.02.003)
Supplement: Supplmental Material [file NIHMS1893289-supplement-Supplmental_Material.pptx]

## Slide 1
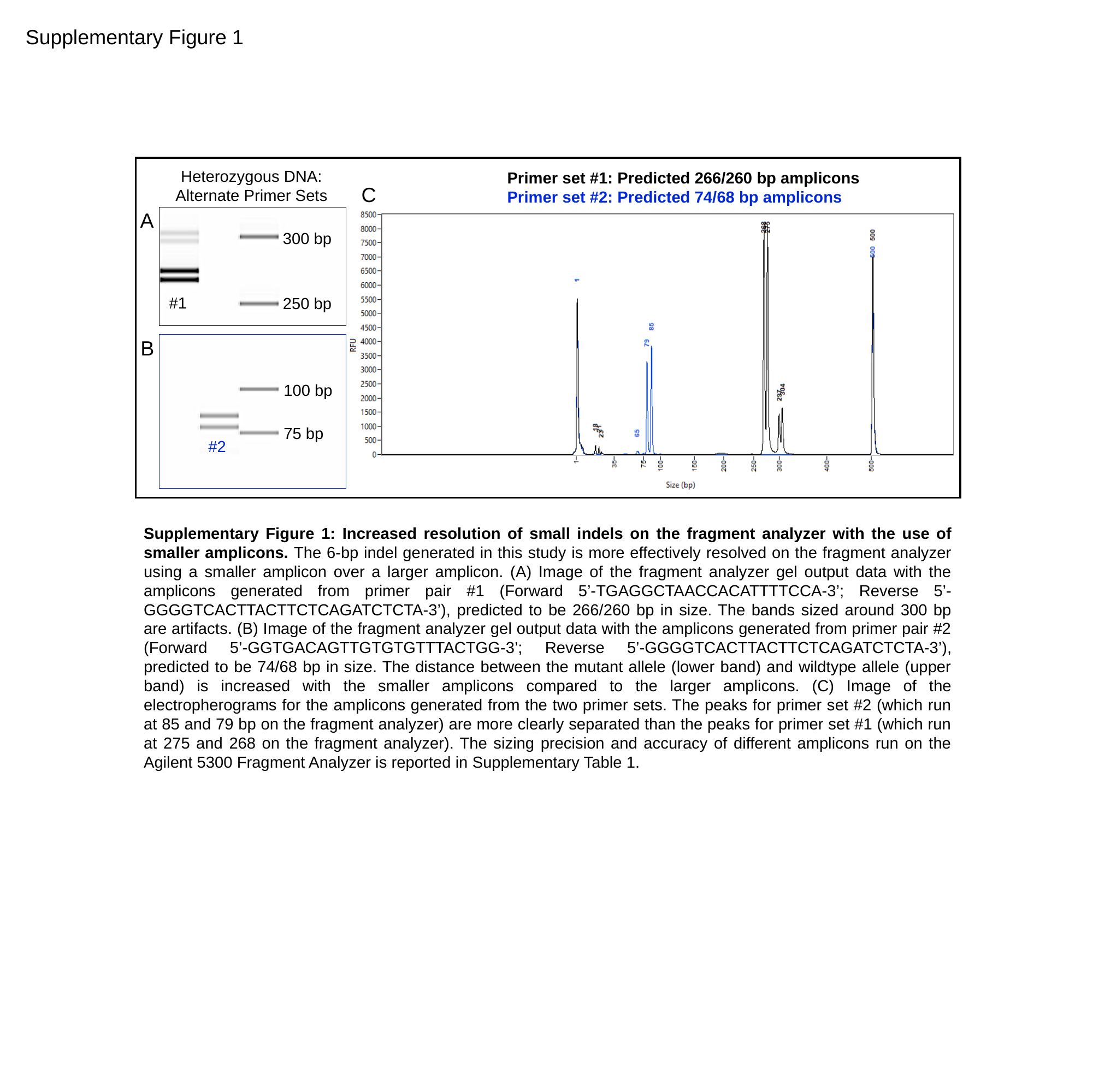

Supplementary Figure 1
Heterozygous DNA:
Alternate Primer Sets
Primer set #1: Predicted 266/260 bp amplicons
Primer set #2: Predicted 74/68 bp amplicons
C
A
300 bp
#1
250 bp
B
100 bp
75 bp
#2
Supplementary Figure 1: Increased resolution of small indels on the fragment analyzer with the use of smaller amplicons. The 6-bp indel generated in this study is more effectively resolved on the fragment analyzer using a smaller amplicon over a larger amplicon. (A) Image of the fragment analyzer gel output data with the amplicons generated from primer pair #1 (Forward 5’-TGAGGCTAACCACATTTTCCA-3’; Reverse 5’-GGGGTCACTTACTTCTCAGATCTCTA-3’), predicted to be 266/260 bp in size. The bands sized around 300 bp are artifacts. (B) Image of the fragment analyzer gel output data with the amplicons generated from primer pair #2 (Forward 5’-GGTGACAGTTGTGTGTTTACTGG-3’; Reverse 5’-GGGGTCACTTACTTCTCAGATCTCTA-3’), predicted to be 74/68 bp in size. The distance between the mutant allele (lower band) and wildtype allele (upper band) is increased with the smaller amplicons compared to the larger amplicons. (C) Image of the electropherograms for the amplicons generated from the two primer sets. The peaks for primer set #2 (which run at 85 and 79 bp on the fragment analyzer) are more clearly separated than the peaks for primer set #1 (which run at 275 and 268 on the fragment analyzer). The sizing precision and accuracy of different amplicons run on the Agilent 5300 Fragment Analyzer is reported in Supplementary Table 1.

## Slide 2
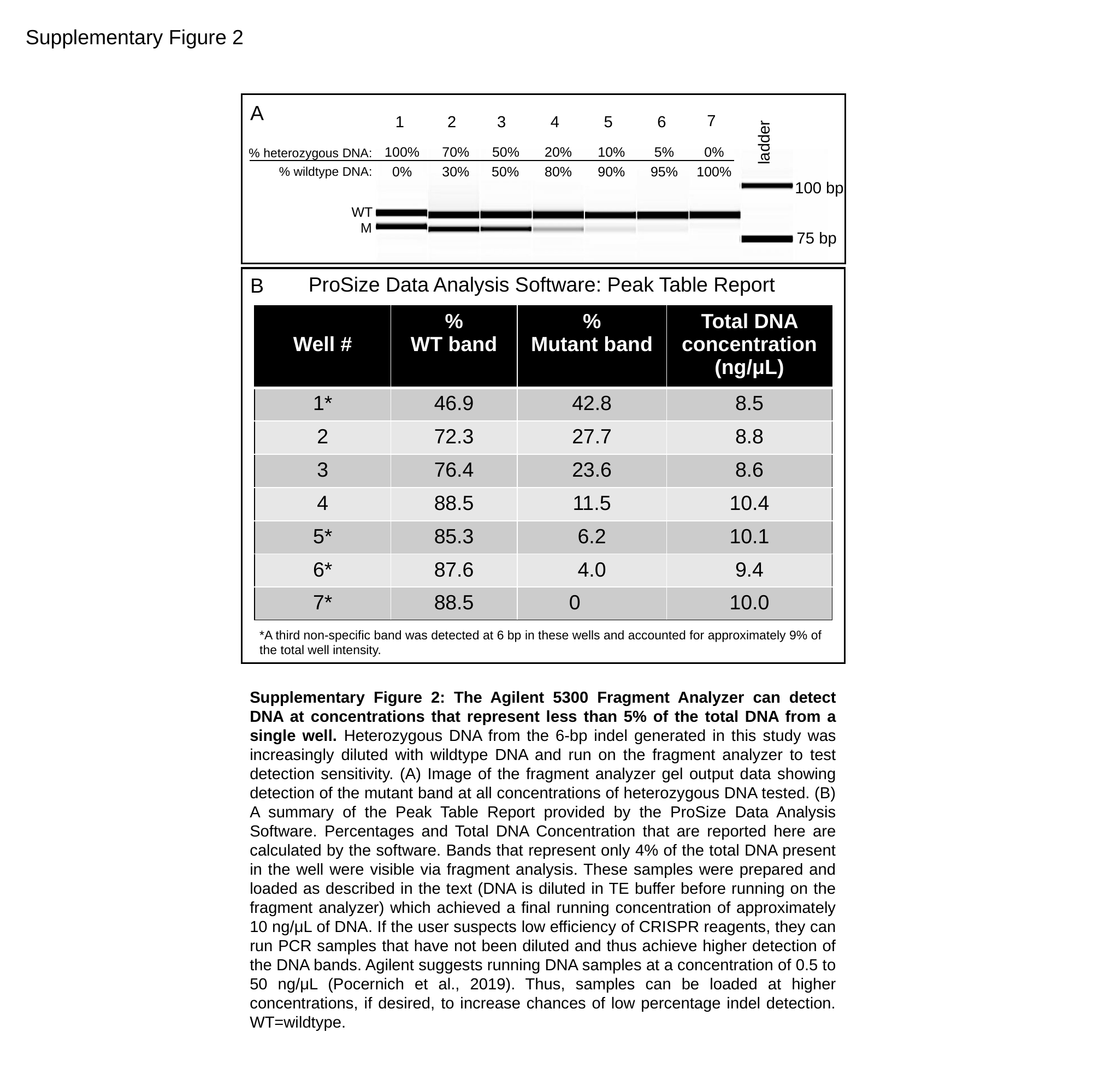

Supplementary Figure 2
A
7
1
2
3
4
5
6
ladder
100%
70%
50%
20%
10%
5%
0%
% heterozygous DNA:
0%
30%
50%
80%
90%
95%
100%
% wildtype DNA:
100 bp
WT
M
75 bp
ProSize Data Analysis Software: Peak Table Report
B
| Well # | % WT band | % Mutant band | Total DNA concentration (ng/μL) |
| --- | --- | --- | --- |
| 1\* | 46.9 | 42.8 | 8.5 |
| 2 | 72.3 | 27.7 | 8.8 |
| 3 | 76.4 | 23.6 | 8.6 |
| 4 | 88.5 | 11.5 | 10.4 |
| 5\* | 85.3 | 6.2 | 10.1 |
| 6\* | 87.6 | 4.0 | 9.4 |
| 7\* | 88.5 | 0 | 10.0 |
*A third non-specific band was detected at 6 bp in these wells and accounted for approximately 9% of the total well intensity.
Supplementary Figure 2: The Agilent 5300 Fragment Analyzer can detect DNA at concentrations that represent less than 5% of the total DNA from a single well. Heterozygous DNA from the 6-bp indel generated in this study was increasingly diluted with wildtype DNA and run on the fragment analyzer to test detection sensitivity. (A) Image of the fragment analyzer gel output data showing detection of the mutant band at all concentrations of heterozygous DNA tested. (B) A summary of the Peak Table Report provided by the ProSize Data Analysis Software. Percentages and Total DNA Concentration that are reported here are calculated by the software. Bands that represent only 4% of the total DNA present in the well were visible via fragment analysis. These samples were prepared and loaded as described in the text (DNA is diluted in TE buffer before running on the fragment analyzer) which achieved a final running concentration of approximately 10 ng/μL of DNA. If the user suspects low efficiency of CRISPR reagents, they can run PCR samples that have not been diluted and thus achieve higher detection of the DNA bands. Agilent suggests running DNA samples at a concentration of 0.5 to 50 ng/μL (Pocernich et al., 2019). Thus, samples can be loaded at higher concentrations, if desired, to increase chances of low percentage indel detection. WT=wildtype.

## Slide 3
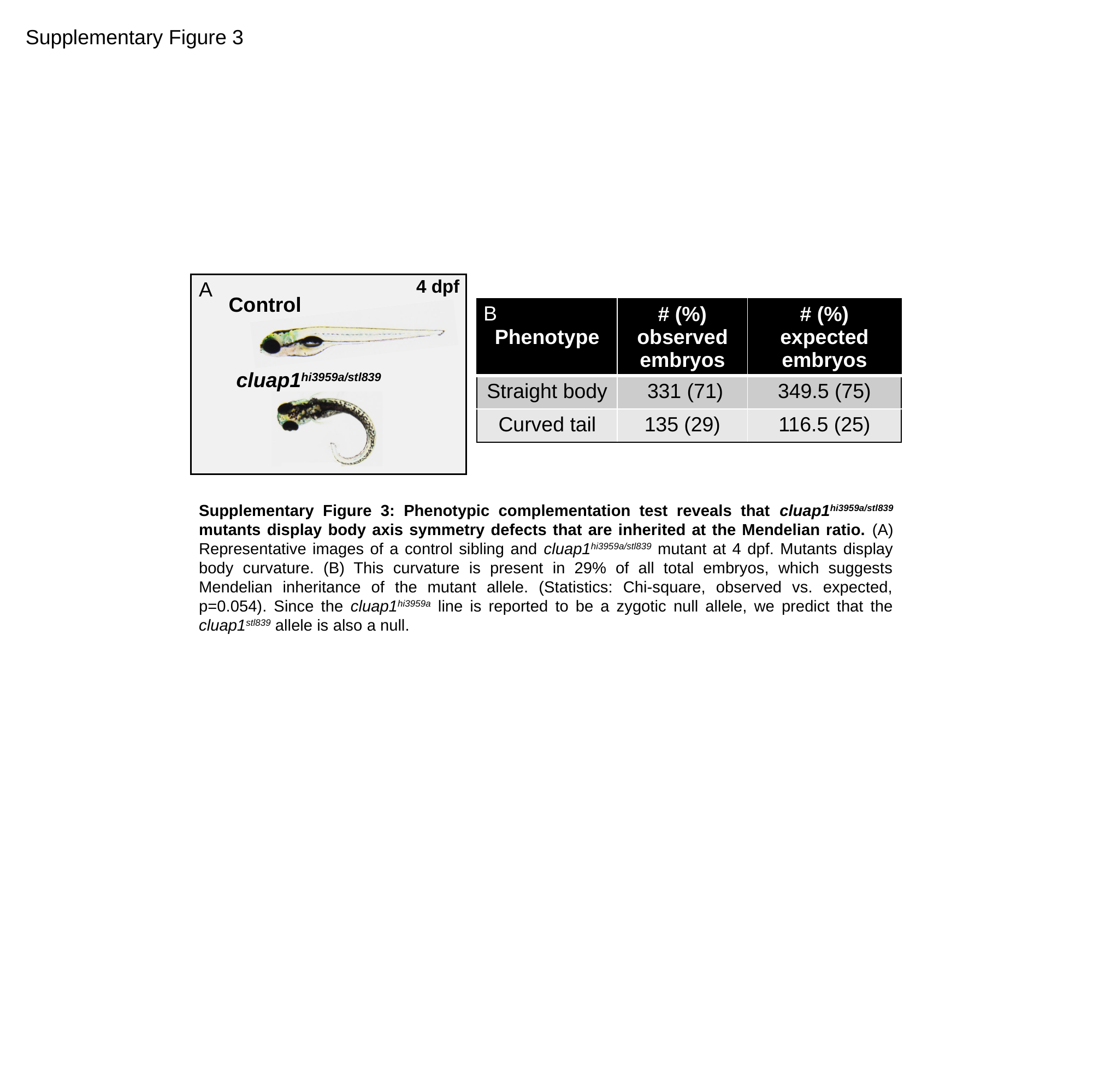

Supplementary Figure 3
4 dpf
A
Control
cluap1hi3959a/stl839
B
| Phenotype | # (%) observed embryos | # (%) expected embryos |
| --- | --- | --- |
| Straight body | 331 (71) | 349.5 (75) |
| Curved tail | 135 (29) | 116.5 (25) |
Supplementary Figure 3: Phenotypic complementation test reveals that cluap1hi3959a/stl839 mutants display body axis symmetry defects that are inherited at the Mendelian ratio. (A) Representative images of a control sibling and cluap1hi3959a/stl839 mutant at 4 dpf. Mutants display body curvature. (B) This curvature is present in 29% of all total embryos, which suggests Mendelian inheritance of the mutant allele. (Statistics: Chi-square, observed vs. expected, p=0.054). Since the cluap1hi3959a line is reported to be a zygotic null allele, we predict that the cluap1stl839 allele is also a null.

## Slide 4
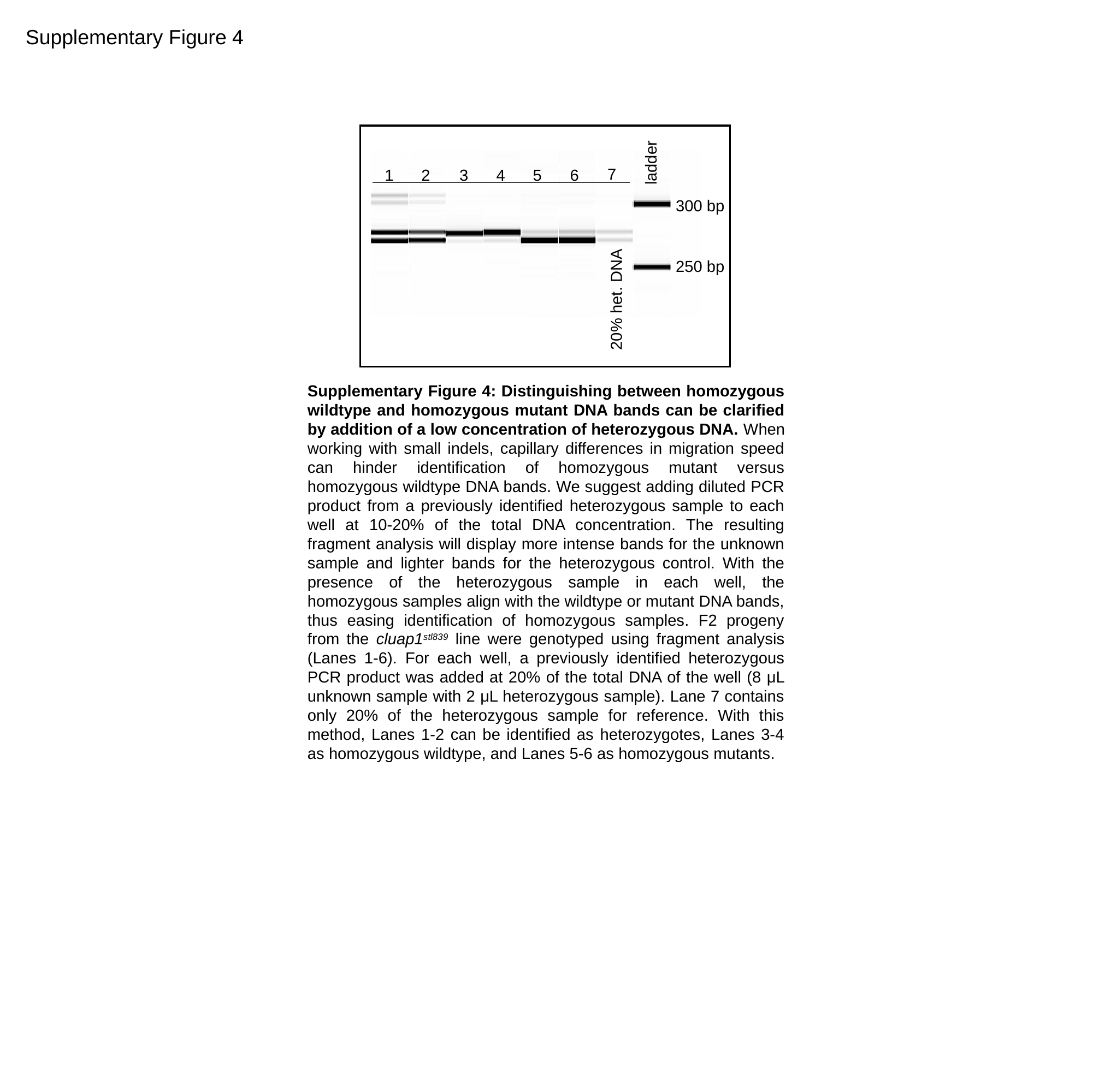

Supplementary Figure 4
ladder
7
1
2
3
4
5
6
300 bp
250 bp
20% het. DNA
Supplementary Figure 4: Distinguishing between homozygous wildtype and homozygous mutant DNA bands can be clarified by addition of a low concentration of heterozygous DNA. When working with small indels, capillary differences in migration speed can hinder identification of homozygous mutant versus homozygous wildtype DNA bands. We suggest adding diluted PCR product from a previously identified heterozygous sample to each well at 10-20% of the total DNA concentration. The resulting fragment analysis will display more intense bands for the unknown sample and lighter bands for the heterozygous control. With the presence of the heterozygous sample in each well, the homozygous samples align with the wildtype or mutant DNA bands, thus easing identification of homozygous samples. F2 progeny from the cluap1stl839 line were genotyped using fragment analysis (Lanes 1-6). For each well, a previously identified heterozygous PCR product was added at 20% of the total DNA of the well (8 μL unknown sample with 2 μL heterozygous sample). Lane 7 contains only 20% of the heterozygous sample for reference. With this method, Lanes 1-2 can be identified as heterozygotes, Lanes 3-4 as homozygous wildtype, and Lanes 5-6 as homozygous mutants.

## Slide 5
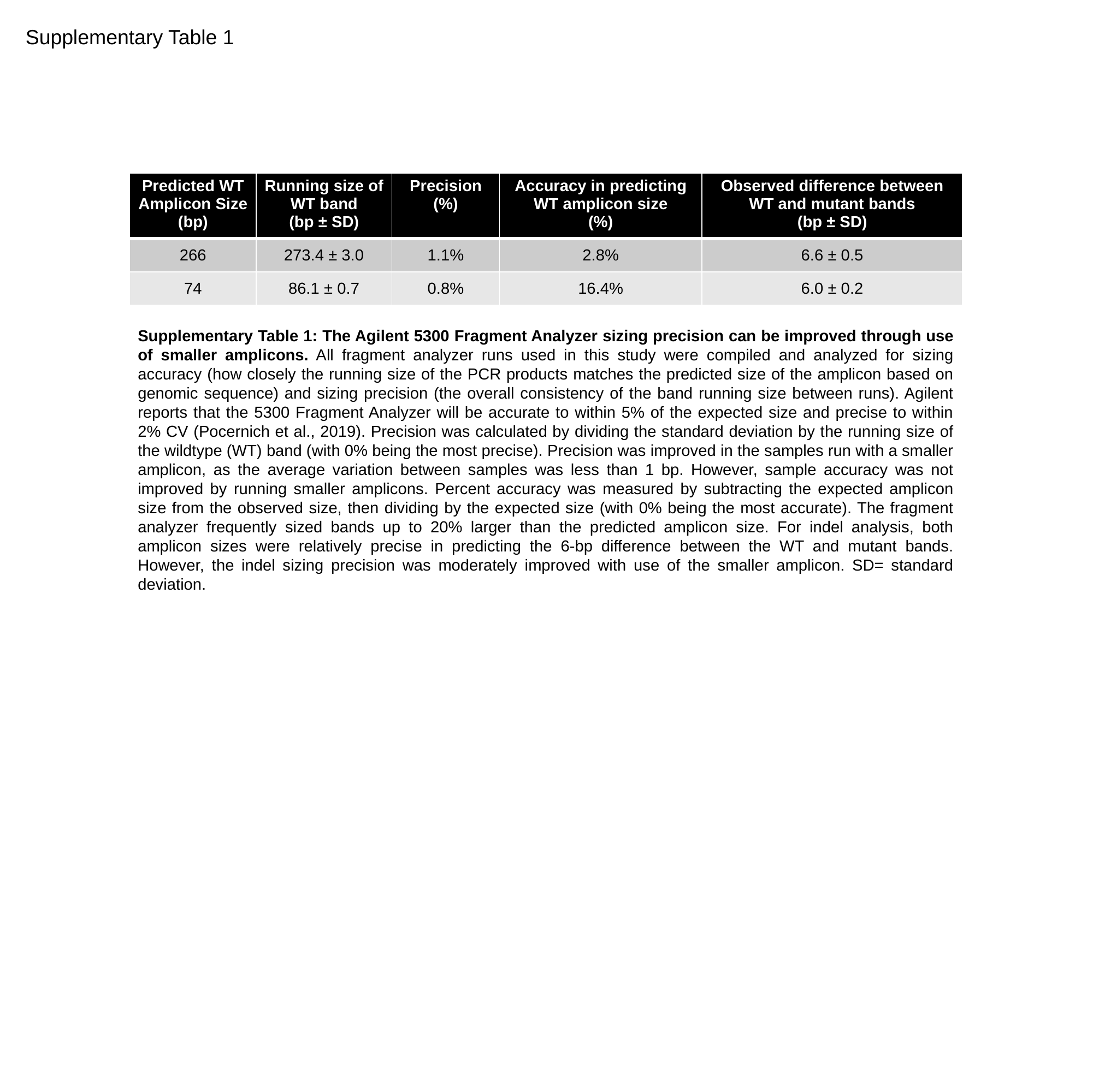

Supplementary Table 1
| Predicted WT Amplicon Size (bp) | Running size of WT band (bp ± SD) | Precision (%) | Accuracy in predicting WT amplicon size (%) | Observed difference between WT and mutant bands (bp ± SD) |
| --- | --- | --- | --- | --- |
| 266 | 273.4 ± 3.0 | 1.1% | 2.8% | 6.6 ± 0.5 |
| 74 | 86.1 ± 0.7 | 0.8% | 16.4% | 6.0 ± 0.2 |
Supplementary Table 1: The Agilent 5300 Fragment Analyzer sizing precision can be improved through use of smaller amplicons. All fragment analyzer runs used in this study were compiled and analyzed for sizing accuracy (how closely the running size of the PCR products matches the predicted size of the amplicon based on genomic sequence) and sizing precision (the overall consistency of the band running size between runs). Agilent reports that the 5300 Fragment Analyzer will be accurate to within 5% of the expected size and precise to within 2% CV (Pocernich et al., 2019). Precision was calculated by dividing the standard deviation by the running size of the wildtype (WT) band (with 0% being the most precise). Precision was improved in the samples run with a smaller amplicon, as the average variation between samples was less than 1 bp. However, sample accuracy was not improved by running smaller amplicons. Percent accuracy was measured by subtracting the expected amplicon size from the observed size, then dividing by the expected size (with 0% being the most accurate). The fragment analyzer frequently sized bands up to 20% larger than the predicted amplicon size. For indel analysis, both amplicon sizes were relatively precise in predicting the 6-bp difference between the WT and mutant bands. However, the indel sizing precision was moderately improved with use of the smaller amplicon. SD= standard deviation.
